# WHO Pandemic Agreement: The need for scientific implementation

**DOI:** 10.1371/journal.ppat.1013729

**Published:** 2025-11-25

**Authors:** Hao Wang, Min Yue

**Affiliations:** 1 Key Laboratory of Systems Health Science of Zhejiang Province, School of Life Science, Hangzhou Institute for Advanced Study, University of Chinese Academy of Sciences, Hangzhou, China; 2 State Key Laboratory for Diagnosis and Treatment of Infectious Diseases, National Clinical Research Center for Infectious Diseases, National Medical Center for Infectious Diseases, The First Affiliated Hospital, College of Medicine, Zhejiang University, Hangzhou, China; Fred Hutchinson Cancer Center, UNITED STATES OF AMERICA

## Introduction

The COVID-19 pandemic acted as a stark diagnostic for the state of global health architecture, revealing a profound inequity exemplified by the persisting vaccination gap by the end of 2023, with coverage reaching nearly 80% in high-income nations while only 30% in low-income countries [1]. As a product of a market-driven distribution model that prioritized profit over people, the disparity did not merely prolong the pandemic; it inflicted millions of avoidable deaths and precipitated a global economic shockwave [2]. The profound crisis of conscience and governance catalyzes the creation of the WHO Pandemic Agreement in May 2025, a legally binding treaty designed to place equity at the center of future pandemic prevention, preparedness, and response [3].

While this accord represents a landmark political achievement, its success will not be guaranteed by signatures alone. Beyond the diplomatic arena, the true test of this treaty lies in the laboratories, data centers, and manufacturing plants where the operational bedrock for the collective aspirations is meticulously constructed. In this perspective, we argue that translating the Pandemic Agreement from text to reality hinges on overcoming three formidable implementation hurdles: (1) the “Pathogen X” readiness, which demands a shift from bespoke R&D to versatile platform technologies capable of addressing unforeseen threats; (2) the integrated surveillance, which requires a transition from reactive reporting to a predictive, global “pathogen Surv-Radar;” and (3) the science of decentralized manufacturing, which necessitates mastering the complexities of global technology transfer and harmonized quality assurance for advanced medical countermeasures. This perspective will deconstruct these barriers and propose a practical roadmap to facilitate the full and robust implementation of the WHO Pandemic Agreement, transforming this historic document into a functional, equitable, and life-saving reality.

## The “Pathogen X” readiness: Beyond predictable threats

The centerpiece of the Agreement, the Pathogen Access and Benefit-Sharing (PABS) system, which mandates a 20% contribution of pandemic products in exchange for pathogen access [4], is predicated on a linear model: a pathogen emerges, a product is developed, and then it is distributed. The scientific reality, however, is far more complex. The PABS system is probably ill-equipped to handle the fundamental uncertainty of “Pathogen X,” a placeholder for a threat whose biological characteristics are unknown [5]. Unlike influenza, where decades of research have yielded relatively predictable vaccine platforms, Pathogen X could be a novel paramyxovirus with unique cell entry mechanisms, an airborne filovirus requiring BSL-4 handling, or even a drug-resistant fungus demanding entirely new therapeutic modalities. The current R&D ecosystem, driven by commercial incentives, is structured to produce bespoke solutions for known diseases, not to maintain a state of agile readiness for the unknown.

This creates a scientific and ethical dilemma. Without a fundamental reorientation of R&D, the PABS system risks becoming an equity mechanism for only those diseases that align with high-income market interests. Pathogens that primarily threaten Low- and Middle-Income Countries (LMICs) may never even enter into the development stage, rendering the PABS distribution mechanism moot.

### Proposed solution: A global “platform technology toolbox” and mission-driven R&D

The solution lies in shifting from a product-centric to a platform-centric R&D model, managed as a global public good.

#### A publicly-funded platform library for plug-and-play.

International funders such as Coalition for Epidemic Preparedness Innovations [6] and the new Pandemic Fund must pivot investment toward developing, validating, and licensing a diverse library of platform technologies. This includes not only mRNA, adjuvant, viral vectors, and host-directed therapies, but also next-generation technologies, including self-amplifying RNA, nanoparticle-based vaccines, broadly acting monoclonal antibodies, and host-directed therapies. By maintaining such a diverse and validated “technology toolbox” in a state of readiness, the scientific community can bypass the need to start from a “zero base” when Pathogen X emerges. Researchers can rapidly select platforms—mRNA, viral vector, or nanoparticle—that best matche the new threat’s biology. As long as the specific antigenic information is obtained and then swiftly integrated into the pre-validated backbone, this plug-and-play modular enables the rapid initiation of candidate vaccine and therapeutic manufacturing.

#### Pre-negotiated, legally binding technology transfer.

The Agreement’s call for technology transfer must be operationalized through pre-negotiated, legally binding agreements. These “dormant” contracts, activated during a public health emergency, would detail the exact terms for transferring not just patents, but the critical, often unpatented, know-how: cell line development protocols, purification chromatography techniques, and adjuvant formulation recipes. This moves beyond vague promises to a concrete, actionable mechanism.

#### A global clinical trial network.

A permanent, WHO-coordinated global clinical trial network, with pre-approved master protocols and established sites in Africa, Asia, and Latin America, is essential. This would ensure that when a new candidate product emerges from a platform, it can be evaluated for safety and efficacy in diverse populations simultaneously, collapsing the development timeline from years to months. Establishment of this global network can be effectively facilitated by integrating with existing infrastructures—such as the HIV Vaccine Trials Network (https://www.hvtn.org/)—that possess extensive experience in multi-site coordination, community engagement, and the implementation of standardized research protocols.

## The integrated surveillance: Moving away from reactive reporting toward proactive intelligence

The Agreement’s symbiosis with the International Health Regulations—where data sharing is incentivized by access to countermeasures—is theoretically powerful. The effectiveness of data sharing largely depends on the quality and nature of the data. However, the scientific obstacle is that our current global surveillance system is largely reactive and fragmented. It is designed to report unusual clusters of human disease, which often serve as a lagging indicator of a much larger, silent outbreak. It fails to systematically integrate the vast streams of data from animal health and environmental sources that are critical for predicting risk before it spills over into human populations. The cryptic transmission of Invasive Non-Typhoidal *Salmonella* in China, uncovered only through advanced genomic epidemiology, is a case in point: a significant public health threat can circulate undetected by traditional surveillance for years [7–9].

Asking a country to share sensitive pathogen data requires immense trust—trust that was shattered during COVID-19 when early reporters were penalized with travel bans [10]. Without a system that provides tangible, predictive value back to the reporting country, data sharing will be seen as an act of risk, not a collaborative benefit.

### Proposed solution: A global pathogen “Surv-Radar”

A global surveillance system that functions less like a fire alarm and more like a sophisticated radar must be built to provide predictive intelligence.

#### Fusing multi-omics data streams.

Operationalizing the “One Health” concept requires a global data platform that integrates genomic, transcriptomic, and proteomic data from diverse sources, including wastewater, livestock, wildlife, and human clinical settings [11,12]. The deadliest threats arise from how a pathogen interacts with its host. Traditional surveillance answers what is present; a multi-omics approach reveals how it behaves. For instance, such integrated data could reveal not just the presence of a potentially dangerous bacterium, but, more critically, that it has actively switched on genes for toxin production, antibiotic resistance, and pathogen–host interactions. By applying AI and machine learning models to integrate and analyze these multi-omics data streams from sources, this platform could detect subtle but significant anomalous patterns—such as the emergence of a new viral genotype in bats coupled with molecular signatures of host adaptation in a high-risk interface zone—flagging the threat for heightened alert long before it appears in human clinics.

#### Building “interpretation capacity,” not just infrastructure.

The focus of global investment must shift from simply supplying sequencers to building end-to-end bioinformatics capacity in LMICs. This includes training a cadre of “genomic epidemiologists,” establishing local data analysis pipelines, and ensuring robust data governance. Without the sovereign capability to analyze and interpret their own data, countries will remain passive providers rather than active partners in global health security.

#### An independent scientific risk assessment body.

To de-politicize the declaration of a Public Health Emergency of International Concern, an independent scientific advisory group should be established under the auspices of the WHO [13]. Comprised of microbiologists, epidemiologists, and public health experts, this body would be responsible for assessing threats based on transparent scientific criteria, providing an impartial recommendation to the Director-General and thereby insulating the process from diplomatic pressures.

## The manufacturing science: Decentralization and standardization for equitable access

While the Agreement’s vision for a Global Supply Chain and Logistics Network rightly aims to dismantle the deadly manufacturing monopolies of the last pandemic [14], its ambition confronts an ultimate practical test in the physical world. The goal of establishing a decentralized, regional manufacturing network is laudable, but it moves beyond policy into the unforgiving realm of manufacturing science. The challenge transcends simply financing and building factories; it lies in mastering the science of precise replication. Ensuring a complex biologic like an mRNA vaccine, produced in a new facility in South Africa, is perfectly bioequivalent—in its molecular integrity, purity, efficacy, and stability—to one from a state-of-the-art facility in Germany is perhaps the most tangible and formidable hurdle the Agreement faces. This is a domain where theory ends and engineering begins, where subtle deviations in raw material sourcing or even ambient temperature can have profound and irreversible impacts on the final product.

This challenge also carries a profound ethical weight. If regional products are perceived as being of lower quality, it could exacerbate vaccine hesitancy and undermine trust in both local and global health authorities. “Good enough” is not good enough when lives are at stake.

### Proposed solution: A network of global biomanufacturing excellence and open science

#### Complete “copy-and-paste” tech transfer packages.

The WHO, in partnership with industry and academia, should coordinate the development of complete “tech transfer packages” for key platform technologies. These would include not just blueprints and protocols, but also standardized training modules, virtual reality-based simulations for complex procedures, and lists of pre-qualified equipment and raw material suppliers.

#### A global network of biomanufacturing centers of excellence.

Regional hubs, such as those being developed in Rwanda and Senegal, should be designated as “Centers of Excellence” in Africa [15]. As an example for other regions worldwide, their role would be twofold: to act as regional training centers for the workforce and to serve as reference laboratories for harmonizing quality control standards across the continent.

#### Open-source quality control methods.

To ensure consistency and build trust, a library of open-source, validated analytical methods for assessing product quality (e.g., measuring mRNA integrity, lipid nanoparticle size, and protein aggregation) should be established. This empowers national regulatory authorities with the tools to independently verify the quality of locally produced countermeasures, fostering transparency and mutual recognition of standards.

## Conclusion

The WHO Pandemic Agreement represents a historic political compact, a promise to future generations that the catastrophic failures of COVID-19 will not be repeated. It reflects a shared recognition of a Community of Shared Future for Mankind that no one is safe until everyone is safe. However, for this promise to be realized, it must be translated from a political aspiration into a scientific blueprint—a shared global commitment to constructing the technical, intellectual, and ethical pillars of genuine preparedness.

The path forward demands key recalibrations in the practice of global health science—moving from a reactive to a predictive posture in surveillance, from a product-focused to a platform-based approach in R&D, and from a centralized to a distributed, quality-assured model in manufacturing. These are not incremental adjustments; they are systematic shifts that challenge entrenched commercial interests and demand unprecedented levels of open collaboration. Critically, the success of any scientific implementation is ultimately dependent on public trust and acceptance. Our responsibility does not end with the production of robust evidence. We must also actively communicate that evidence, engaging directly with diverse publics and translating complex scientific findings into clear, reliable, and accessible information.
